# Human *FasL* Gene Is a Target of β-Catenin/T-Cell Factor Pathway and Complex *FasL* Haplotypes Alter Promoter Functions

**DOI:** 10.1371/journal.pone.0026143

**Published:** 2011-10-11

**Authors:** Jianming Wu, Maureen H. Richards, Jinhai Huang, Lena Al-Harthi, Xiulong Xu, Rui Lin, Fenglong Xie, Andrew W. Gibson, Jeffrey C. Edberg, Robert P. Kimberly

**Affiliations:** 1 Department of Veterinary and Biomedical Sciences, University of Minnesota, St. Paul, Minnesota, United States of America; 2 Department of Immunology and Microbiology, Rush Presbyterian St. Luke's Medical Center, Chicago, Illinois, United States of America; 3 Department of General Surgery, Rush Presbyterian St. Luke's Medical Center, Chicago, Illinois, United States of America; 4 Division of Clinical Immunology and Rheumatology, University of Alabama at Birmingham, Birmingham, Alabama, United States of America; Northwestern University Feinberg School of Medicine, United States of America

## Abstract

*FasL* expression on human immune cells and cancer cells plays important roles in immune homeostasis and in cancer development. Our previous study suggests that polymorphisms in the *FasL* promoter can significantly affect the gene expression in human cells. In addition to the functional *FasL* SNP -844C>T (rs763110), three other SNPs (SNP -756A>G or rs2021837, SNP -478A>T or rs41309790, and SNP -205 C>G or rs74124371) exist in the proximal *FasL* promoter. In the current study, we established three major FasL hyplotypes in humans. Interestingly, a transcription motif search revealed that the *FasL* promoter possessed two consensus T-cell factor (TCF/LEF1) binding elements (TBEs), which is either polymorphic (SNP -205C>G) or close to the functional SNP -844C>T. Subsequently, we demonstrate that both *FasL* TBEs formed complexes with the TCF-4 and β-catenin transcription factors *in vitro* and *in vivo*. Co-transfection of LEF-1 and β-catenin transcription factors significantly increased *FasL* promoter activities, suggesting that *FasL* is a target gene of the β-catenin/T-cell factor pathway. More importantly, we found that the rare allele (-205G) of the polymorphic *FasL* TBE (SNP -205C>G) failed to bind the TCF-4 transcription factor and that SNP -205 C>G significantly affected the promoter activity. Furthermore, promoter reporter assays revealed that *FasL* SNP haplotypes influenced promoter activities in human colon cancer cells and in human T cells. Finally, β-catenin knockdown significantly decreased the *FasL* expression in human SW480 colon cancer cells. Collectively, our data suggest that β-catenin may be involved in *FasL* gene regulation and that FasL expression is influenced by *FasL* SNP haplotypes, which may have significant implications in immune response and tumorigenesis.

## Introduction

FasL (Fas ligand or CD95 ligand) is a type II membrane protein and a member of the TNF ligand superfamily. FasL is mainly expressed in activated T cells, NK cells, macrophages, and various cancer cells. FasL triggers cell death and/or cell activation by binding and clustering Fas (CD95). Fas-mediated apoptosis or activation induced cell death (AICD) plays important roles in maintaining peripheral immune tolerance [1]. In mouse models, the *gld* and *lpr* mice containing the loss-of-function mutations in the *FasL* (*gld* mice) and *Fas* (*lpr* mice) genes develop spontaneous autoimmunity as a result of defective lymphocyte apoptosis [2], [3]. The FasL/Fas system promotes immune tolerance through the deletion of auto-reactive T cells, B cells, and macrophages [4]–[6]. In humans, autoimmune lymphoproliferative syndrome (ALPS or Canale-Smith syndrome) is caused by the inherited loss-of-function mutations in *Fas* or *FasL* [7]-[9]. FasL also initiates cell activation and cell differentiation by engaging Fas, which promotes chronic inflammation and inflammatory responses [10], [11]. Furthermore, FasL has a critical role in the pathogenesis of AIDS and in the induction of pulmonary silicosis [12]–[14].

The FasL/Fas system is essential to establish and maintain immune privilege for organs or tissues [15], [16]. Most notably, FasL is widely expressed in various human cancers such as melanoma, hepatocellular carcinoma, lung cancer, astrocytoma, esophageal carcinoma, gastric adenocarcinomas, ovarian carcinoma, and colon adenocarcinomas [17]–[29]. FasL-expressing tumor cells kill the tumor-infiltrating lymphocytes (TILs) through Fas-mediated apoptosis, which is considered as the most important tool that cancer cells use to counterattack the human immune system [30]. Furthermore, the Fas/FasL system was found to have a growth-promoting role during tumorigenesis, highlighting that the cancer cells expressing both FasL and Fas may have the growth advantage through autocrine signaling [31]. Several studies demonstrated that FasL expression on cancer cells facilitates the establishment of tumor metastases [29], [32], [33]. Accumulating genetic evidence also supports a role for FasL in cancer development because the polymorphisms in *FasL* or *Fas* are associated with cancer risks [34]. Collectively, FasL seems to play a critical role in tumorigenesis and tumor metastasis [19]–[27], [30], [35], [36].

The expression of FasL is tightly controlled in humans. Numerous transcription factors are involved the regulation of the *FasL* gene. NF-κB, NF-AT, Egr-3, and IRF-1 have been implicated in regulation of human *FasL* gene [37]–[43]. Previously, we reported that a polymorphic C/EBPβ element in the *FasL* promoter region is involved in the regulation of FasL expression [44]. In the current study, we characterized two TCF/LEF-1 binding elements (TBEs) in the *FasL* promoter region. We demonstrate that *FasL* TBEs are involved in the regulation of promoter activity. Additionally, we show that *FasL* SNP haplotypes affected the promoter activities in human cells. Furthermore, our data support that β-catenin is involved in the regulation of FasL expression in cancer cells. Our study provides new insight into the genetics of human *FasL* and the mechanisms of *FasL* gene regulation in human immune cells and cancer cells.

## Results

### FasL SNPs and SNP haplotypes

**Figure 1:**
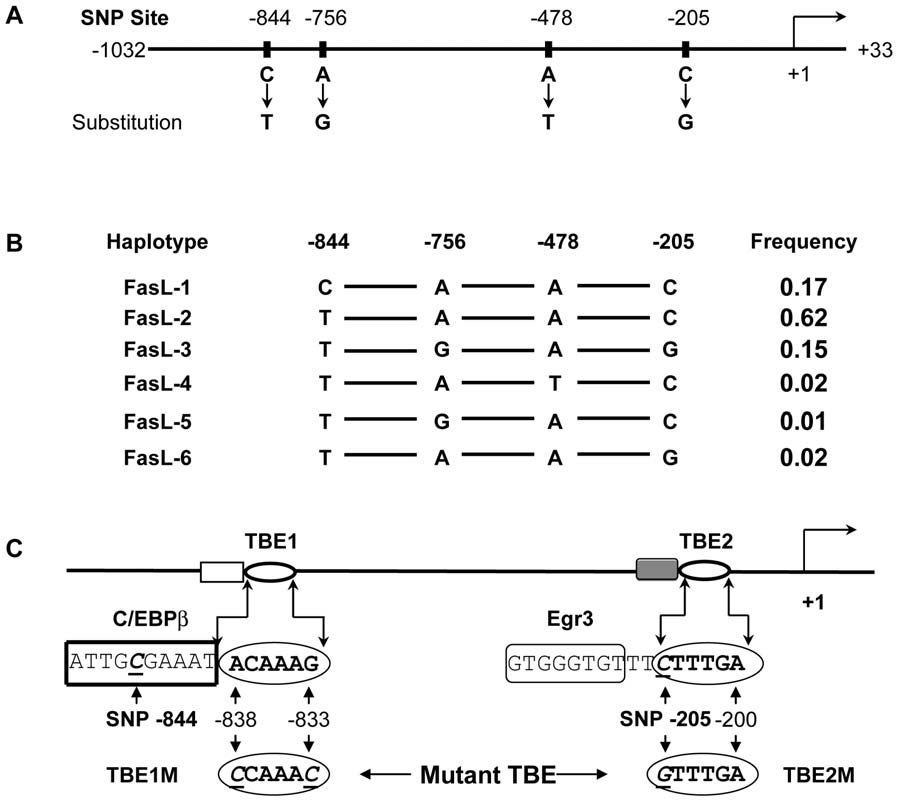
*FasL* promoter SNP haplotypes and location of TCF/LEF-1 binding elements. **A**). Four SNPs (-844C>T, -756A>G, -478A>T, and -205C>G) were identified in the FasL promoter region. **B**). PHASE program for haplotype reconstruction was used for haplotype analysis from 150 African American donors. The *FasL* promoter SNP haplotypes were verified from homozygous donors and further confirmed by sequencing genomic DNA clones from heterozygous donors. The *FasL* haplotype frequencies were calculated according to the established haplotypes. **C**). Identification of TCF/LEF-1 binding elements (TBEs) in the *FasL* promoter region. The core sequence of putative distal TCF/LEF-1 element (TBE1) is between nucleotide position -838 to -833 while the core sequence of the putative proximal TCF/LEF-1 element (TBE2) is between nucleotide position -205 and -200. SNP -205C>G is located within the TBE2 core sequence. The nucleotide changes (underlined) were the introduced mutations in the EMSA probes or in the promoter reporter constructs.

Four SNPs (-844C>T, -756A>G, -478A>T, and -205C>G) were identified in the *FasL* promoter region (Fig. 1A). We were able to construct six *FasL* promoter haplotypes by using PHASE software (www.stat.washington.edu/stephens/software.html) basedon genetic data of 150 African American donors (Fig. 1B). We further confirmed the *FasL* SNP haplotypes by sequencing multiple genomic DNA clones from six heterozygous donors. Three major *FasL* promoter haplotypes (FasL-1, FasL-2, and FasL-3) were identified (Fig. 1B). FasL-2 (-844T/-756A/-478A/-205C) is the most common haplotype with the allele frequency of 0.62. FasL-1 (-844C/-756A/-478A/-205C) is also a common haplotype (allele frequency  =  0.17). As the third major haplotype, FasL-3 (-844T/-756G/-478A/-205G) has the allele frequency of 0.15. All the rare *FasL* haplotypes (FasL-4, FasL-5, and FasL-6) have the gene frequencies less than 0.03 in the population (Fig. 1B).

Subsequently, we carried out transcription factor motif search to look for the potential transcription elements near the *FasL* polymorphic sites. Surprisingly, we found that a putative TCF/LEF-1 binding element (TBE) is adjacent to the SNP -844C>T and that the second putative TBE sits on the *FasL* SNP -205C>G (Fig. 1C). For simplicity, we designated the putative TBE near the SNP -844C>T as TBE1 and the TBE containing the SNP -205C>G as TBE2 (Fig. 1C). Mutant *FasL* TBEs were generated by site-directed mutagenesis in the wild-type TBEs of the promoter reporter constructs (Fig. 1C).

### Characterization of the distal TCF/LEF-1 binding element (TBE1)

**Figure 2:**
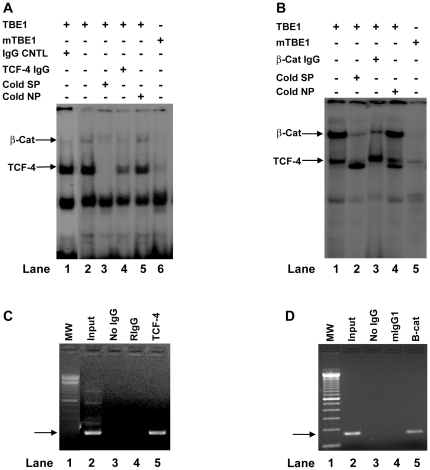
Binding of TCF-4 and β-catenin to the distal *FasL* TCF/LEF-1 binding element (TBE1). **A**). Radio-labeled wild-type TBE1 probe (lane 1-5) and mutant TBE1 probe (lane 6) were incubated with 8 µg of SW480 cell nuclear extracts for 30 min. Competition experiments were performed by preincubating with 200 fold molar excess of the unlabeled TBE1 probes (Cold SP, lane 3), or non-specific probe (Cold NP, lane 5). Antibody binding experiments were carried out following the vendor's instruction with anti-TCF-4 antibody (lane 4) and rabbit IgG as control (lane 1). The arrow indicates the position of specific transcription factor complexes. Results shown were representative of four experiments. **B**). Radio-labeled TBE1 (lane 1-4) and mutant TBE1 probes (lane 5) were incubated with 8 µg of Jurkat cell nuclear extracts for 30 min. Antibody binding experiments were carried out with anti-β-catenin antibody (lane 3). The arrows indicate the position of specific transcription factor complexes. Results shown are representative of four experiments. **C**). TCF-4 bound to the TBE1 of *FasL* promoter in a Chromatin Immunoprecipitation Assay (ChIP). ChIP assay was performed as described in “Materials and Methods”. Rabbit anti-human TCF-4 antibody was used to precipitate Jurkat T cell chromatin complexes containing *FasL* promoter DNA fragment (lane 5). The appropriate positive (lane 2) and negative controls (lane 3 and 4) were included. Lane 1 contained DNA molecular weight marker (100 bp DNA ladders). **D**). β-catenin bound to the *FasL* promoter TBE1 in a ChIP assay. Mouse monoclonal antibody against human β-catenin was used to precipitate Jurkat T cell chromatin complexes containing *FasL* promoter DNA fragment (lane 5). The appropriate positive (lane 2) and negative controls (lane 3 and 4) were included. Lane 1 contained DNA molecular weight marker (100 bp DNA ladders). The positive PCR products were shown as 163 bp DNA bands (pointed by arrow) in ChIP assays. The identity of DNA band was further confirmed with DNA sequencing.

The TCF/LEF-1 transcription factor members belong to the HMG (high mobility group) class of transcription regulators that have a highly conserved consensus recognition core motif, 5′-CTTTG(A/T)-3′ [45]. As shown in Fig. 1C, the nucleotide sequence between position -838 and -833 (^-838^ACAAAG^-833^) has a perfect match with the consensus T-cell factor (TCF/LEF-1) binding element (5′-CTTTGT-3′) in the reverse orientation. Therefore, the putative *FasL* TBE1 (the distal TBE) is located next to the C/EBPβ element and five nucleotides away from the SNP -844C>T. To determine whether the putative distal TBE (TBE1) could bind the specific transcription factors, we carried out electrophoretic mobility shift assays (EMSA). As shown in Fig. 2A, complexes were formed between the radio-labeled TBE1 probe and proteins in SW480 nuclear extracts (Lane 1, 2, and 5). The unlabeled TBE1 Specific Probe (cold SP) inhibited the complex formation between the radio-labeled probe and the nuclear extract (Fig. 2A, Lane 3). The anti-TCF-4 antibody significantly decreased the specific complex formation between nuclear proteins and TBE1 probe (Fig. 2A, Lane 4), providing evidence that TCF-4 is one of the transcription factors that binds the TBE1. In addition, cold Non-Specific probe (cold NP) failed to inhibit the complex formation between the labeled TBE1 probe and nuclear proteins (Fig. 2A, Lane 5) and the mutations within the putative TBE1 binding core sequence abrogated the binding of the transcription factors (Lane 6).

In Wingless/Wnt signaling pathways, β-catenin could form complexes with nuclear TCFs to regulate gene expression [46]. To confirm whether the TBE1 probe is capable of forming super complexes with TCFs and β-catenin, we performed EMSAs using anti β-catenin antibody. Fig. 2B shows that radio-labeled TBE1 probe could form complexes with TCF-4 and β-catenin (Lane 1 and 4) and the unlabeled TBE1 probe inhibits the specific complex formation (Lane 2). Furthermore, anti β-catenin antibody dramatically decreased the formation of the probe-TCF-4-β-catenin complexes (β-Cat arrow-pointed band, Fig. 2B, Lane 3). Meanwhile, the probe-TCF-4 complex formation was notably increased (TCF-4 arrow-pointed band, Fig. 2B, Lane 3), confirming that super complexes could be formed between TBE1 probe and TCF-4 plus β-catenin. Mutations within the TBE1 core sequence disrupted the binding of both TCF-4 and β-catenin (Fig. 2A, Lane 6 and Fig. 2B, Lane 5). Taken together, the putative TBE1 adjacent to the SNP -844 is indeed a TCF/LEF-1 transcription factor-binding element.

EMSAs provided direct evidence for the physical interaction between TCF-4 and the TBE1 probes *in vitro*. Next, we performed chromatin immunoprecipitation assays (ChIP) to confirm the *in vivo* interaction between the TBE1 and transcription factors (TCF-4 and β-catenin). As shown in Fig. 2C, anti TCF-4 antibody was able to specifically precipitate chromatin-DNA complexes containing TBE1 DNA fragment (Lane 5). In addition, anti β-catenin antibody was also able to precipitate chromatin-DNA complexes containing TBE1 DNA fragment (Fig. 2D, Lane 5). The identity of DNA fragments in the ChIP assay was further confirmed by direct DNA sequencing (data not shown). Our data confirmed that endogenous TCF-4 and β-catenin bound *FasL* TBE1 *in vivo*, consistent with the *in vitro* results in Fig. 2A and 2B.

### Effect of SNP -205 on the binding affinities for TCF/LEF-1 transcription factors

**Figure 3:**
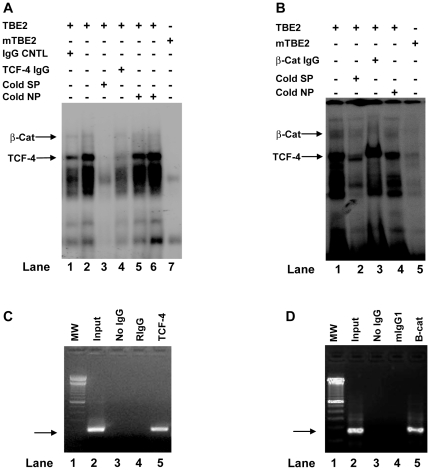
Binding of TCF-4 and β-catenin to the proximal *FasL* TCF/LEF-1 binding element (TBE2). **A**). Radio-labeled wild-type TBE2 probe (-205C allele, lane 1-6) and mutant TBE2 probe (-205G allele) (lane 6) were incubated with 8 µg of SW480 cell nuclear extracts for 30 min. Competition experiments were performed with the unlabeled TBE2 probes (Cold SP, lane 3) or unlabeled non-specific probes (Cold NP, lane 5 and 6). Antibody binding experiments were carried out by using anti-TCF-4 antibody (lane 4) and control rabbit IgG (lane 1). The arrow indicates the position of specific transcription factor complexes. Results shown were representative of four experiments. **B**). Radio-labeled TBE2 probe (lane 1-4) and mutant TBE2 probes (lane 5) were incubated with Jurkat cell nuclear extracts. Competition experiments were performed with the unlabeled TBE2 probe (Cold SP, lane 2), or non-specific probe (Cold NP, lane 4). Antibody binding experiments were carried with anti-β-catenin antibody (lane 3). Arrows indicate the position of specific transcription factor complexes. Results shown are representative of four experiments. **C**). TCF-4 bound to *FasL* TBE2 in a ChIP assay. Anti-human TCF-4 antibody was used to immunoprecipitate Jurkat chromatin complexes containing *FasL* DNA fragment (lane 5). The positive (lane 2) and negative controls (lane 3 and 4) were included. DNA molecular weight marker (100 bp ladders) was in Lane 1. **D**). β-catenin bound to the *FasL* promoter TBE2 in a ChIP assay. Mouse monoclonal antibody against human β-catenin was used to precipitate Jurkat T cell chromatin complexes containing *FasL* promoter DNA fragment (lane 5). The appropriate positive (lane 2) and negative controls (lane 3 and 4) were included. Lane 1 contained DNA molecular weight marker (100 bp DNA ladders). The positive PCR products were shown as 213 bp DNA bands (pointed by arrow) in ChIP assays and the identity of DNA band was further confirmed with DNA sequencing.

Multiple transcription factors (Egr-3, SP-1, and NF-AT) are located near *FasL* SNP -205C>G [38]-[40], [42]. In addition, as *FasL* -205C allele is within the nucleotide sequence (5′-*C* ^-205^TTTGA-3′) that matches perfectly with the consensus TBE (5′-CTTTGA-3′) and therefore, we speculated that the SNP -205C>G may be within a functional TBE (Fig. 1C, TBE2). To examine whether the putative TBE2 is able to bind TCF-4, we carried out EMSAs using the double-stranded DNA probes. Fig. 3A shows that the labeled wild-type TBE2 probe (TBE2, -205C allele) formed complexes with the SW480 nuclear extract proteins (Fig. 3A, Lane 1, 2, 5, and 6). The complex formation was abrogated by the addition of unlabeled wild-type TBE2 probe (cold SP) (Fig. 3A, Lane 3), but the cold non-specific probes (cold NP) were unable to inhibit formation of the specific complexes (Fig. 3A, Lane 5 and 6), validating that the binding of nuclear proteins to the labeled probe was specific. Furthermore, the anti TCF-4 antibody dramatically reduced the complex formation (Fig. 3A, Lane 4), verifying that the transcription factor TCF-4 binds to the TBE2 probe. Interestingly, there was no complex formation between the labeled TBE2 mutant probe (mTBE2 or-205G allele) and TCF-4 (Fig. 3A, Lane 7), suggesting that function of the putative *FasL* TBE2 may be affected by SNP -205 alleles.

We further determined whether the TBE2 probe can form super-complexes with TCF-4 plus β-catenin. Besides the probe-TCF-4 complexes, we were able to detect probe-TCF-4-β-catenin super-complexes (β-Cat arrow-pointed band, Fig. 3B, Lane 1 and 4). Again, the unlabeled TBE2 wild-type probe (cold SP) inhibited the specific complex formation (Fig. 3B, Lane 2). The anti β-catenin antibody was able to disrupt the specific super-complexes containing the labeled probe, TCF-4, and β-catenin (Fig. 3B, Lane 3). With the decrease of probe-TCF-4-β-catenin super-complexes, the probe-TCF-4 complexes increased dramatically (TCF-4 arrow-pointed band, Fig. 3B, Lane 3), verifying that the wild-type TBE2 probe (-205C allele) could form super-complexes with TCF-4 and β-catenin. In contrast, the mutant TBE2 (mTBE2, -205G allele) probe almost lost the ability to bind TCF-4 and β-catenin (Fig. 3B, Lane 5). Taken together, our data indicate that SNP -205C>G is located in a bona fide TCF/LEF-1 transcription factor-binding element and the SNP -205C>G in the TEB2 affect the binding capacity for the transcription factors (TCF-4 and β-catenin). Subsequently, we used chromatin immunoprecipitation assays (ChIP) to examine whether TCF-4 and β-catenin bind to *FasL* TBE2 *in vivo*. As shown in Fig. 3C, anti TCF-4 antibody was able to specifically precipitate chromatin-DNA complexes containing TBE2 DNA fragment (Lane 5). In addition, anti β-catenin antibody was able to specifically precipitate chromatin-DNA complexes containing TBE2 DNA fragment (Fig. 3D, Lane 5). The identity of DNA fragments in the ChIP assay was further confirmed by direct DNA sequencing (data not shown). Our data confirmed that endogenous TCF-4 and β-catenine bound *FasL* TBE2 *in vivo*, which is in agreement with the *in vitro* results shown in Fig. 3A and 3B.

### Up-regulation of FasL promoter activities by human LEF-1 and β-catenin

**Figure 4:**
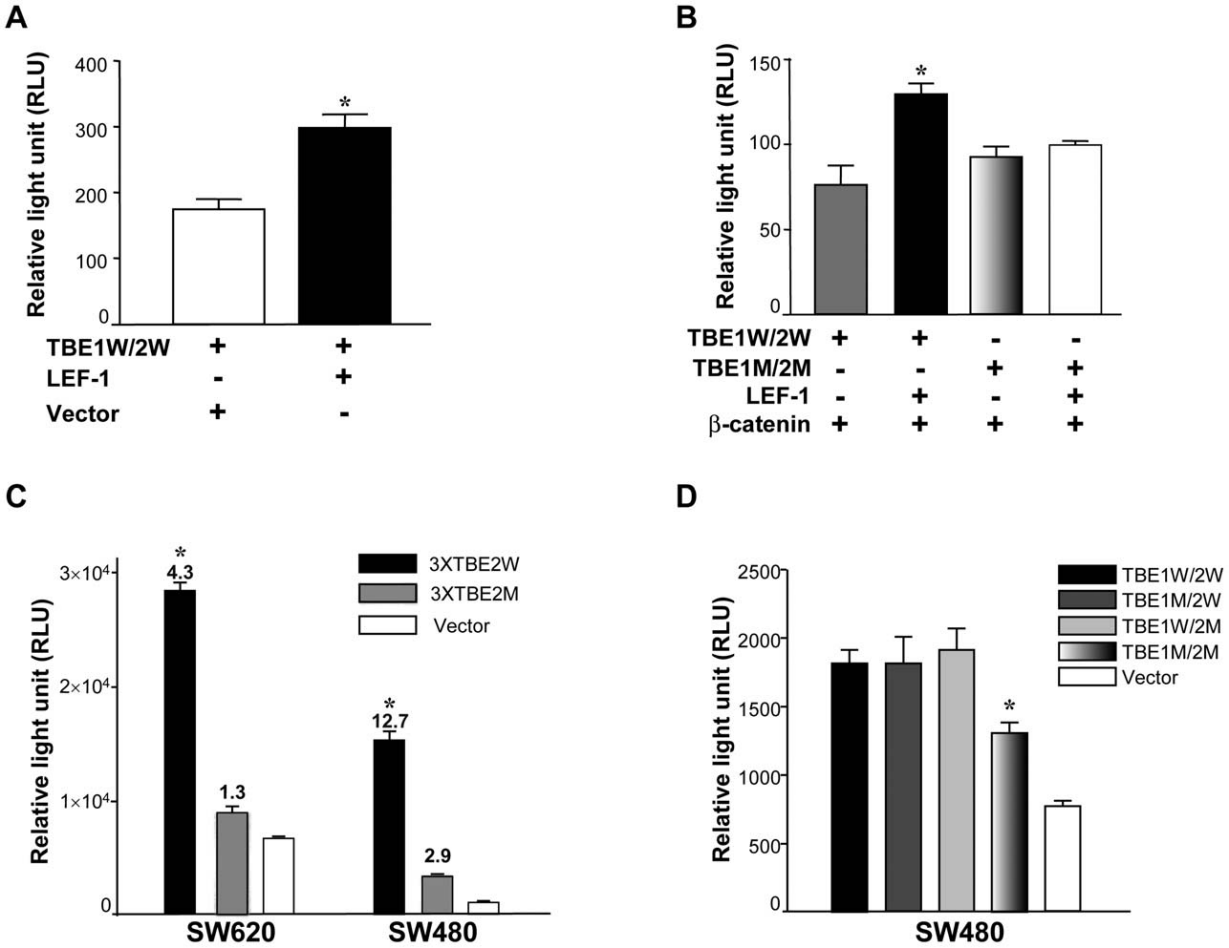
Role of TBEs in *FasL* promoter. **A**). Human LEF-1 increases *FasL* promoter activities in SW480 cells. *FasL* promoter reporter construct (0.5 µg) was co-transfected with human LEF-1 expression construct or vector control plasmid DNA (0.5 µg) into SW480 cells. LEF-1 significantly increased FasL promoter activities compared to the vector DNA control in SW480 cells. Data represent means ± SEM from three independent experiments (*P<0.01).** B**). Functional TBEs are required for the enhancement of *FasL* promoter activity by LEF-1 and β-catenin. *FasL* promoter reporter plasmid DNA (0.5 µg) was co-transfected with LEF-1 (0.5 µg) and β-catenin (1.0 µg) into COS-7 cells. Co-transfection of LEF-1 and β-catenin significantly increased activities of *FasL* promoter containing functional TBEs (TBE1W/2W) in COS-7 cells (*P<0.01). Co-transfection of LEF-1 and β-catenin failed to significantly increase activities of *FasL* promoter containing mutant TBEs (TBE1M/2M). Data represent means ± SEM from three independent experiments. **C**). Promoter reporter plasmid DNA (0.5 µg) containing either triplicate wild-type TBE2 (3×TBE2W, -205C allele) or triplicate mutant TBE2 (3×TBE2M, -205G allele) was transfected into SW480 and SW620 cells as described in “Materials and Methods”. The triplicate wild-type TBE2 (3×TBE2W) significantly increased the promoter activities compared to the triplicate mutant TBE2 (3×TBE2M) in SW620 (4.3 folds for wild-type TBE2 and 1.3 folds for mutant TBE2 over the vector control respectively) and in SW480 (12.7 folds for wild-type TBE2 and 2.9 for mutant TBE2 over the vector control respectively). Data represent means ± SEM from six independent experiments. The star symbol indicates that there are significant differences between 3×TBE2W and 3×TBE2M (*P<0.001). **D**). Role of *FasL* promoter TBEs in SW480 cells. Mutation of either TBE1 (TBE1M/2W) or TBE2 (TBE1W/2M) failed to affect the *FasL* promoter activities compared to the wild-type reporter construct (*P* = 0.50 for TBE1W2M and *P* = 0.30 for TBE1M/2M). Simultaneous mutations of TBE1 and TBE2 (TBE1M/2M) significantly reduced *FasL* promoter activities compared to the construct with wild-type TBE1 and TBE2 (TBE1W/2W) in SW480 cells (*P<0.01). Data represent means ± SEM from four independent experiments. Relative luciferase light units (RLU) were standardized to Renilla luciferase activities (A and B) or standardized to β-galactosidase activities (C and D).

The high mobility group (HMG) domain of TCF/LEF-1 transcription factor family members is responsible for the binding to target genes in a sequence specific fashion while β-catenin provides a domain for transcription activation. The transcriptional activation of target genes occurs only when TCF/LEF-1 transcription factors are associated with β-catenin in cell nuclei. Nuclei of human APC^-/-^ colon cancer cells contain stable β-catenin-TCF-4 complexes for target gene activation [47]. To examine whether *FasL* TBEs are involved in the regulation of *FasL* promoter activities, we carried out promoter reporter assays in human colon cancer SW480 cells, which contain constitutively high levels of active β-catenin in the nuclei [47]. As shown in Fig. 4A, co-transfection of LEF-1 significantly increased promoter activities of the *FasL* reporter construct containing two wild-type TBEs in SW480 cells as compared with the vector control. To further confirm that *FasL* promoter activities could be affected by LEF-1 and β-catenin, we carried out the promoter reporter assays in COS-7 cells. As shown in Fig. 4B, activities of *FasL* promoter containing two wild-type TBEs (TBE1W/2W, left two bars) were significantly increased when cotransfected with LEF-1 and β-catenin. On the other hand, activities of *FasL* promoter containing two mutant TBEs (TBE1M/2M) failed to respond to the co-transfection of LEF-1 and β-catenin (right two bars, Fig. 4B). These data suggest that *FasL* TBEs are involved in the regulation of the *FasL* promoter in human cells and that mutations in *FasL* TBEs affect *FasL* promoter activities.

### Role of FasL TBEs in human colon cancer cells

Because the proximal *FasL* TBE (TBE2) contains the SNP -205C>G, next we examined whether the SNP could affect promoter activities. We carried out the promoter reporter assays with the constructs containing either the triplicate wild-type TBE2 (3XTBE2W, -205C allele) or the triplicate mutant TBE2 (3XTBE2M, -205G allele). As shown in Fig. 4C, triplicate wild-type TBE2 (-205C allele) increased reporter promoter activities four folds in SW620 cells (APC^-/-^ colon cancer cells) and twelve folds in SW480 cells (APC^-/-^ colon cancer cells) over the vector controls respectively. In contrast, triplicate mutant TBE2 (-205G allele) enhanced reporter promoter activities only by 1.3 folds in SW620 cells and by 2.9 folds in SW480 cells over the vector controls. Therefore, wild-type TBE2 (-205C allele) serves as a much better enhancer for *FasL* gene expression and that the SNP -205C>G may affect the *FasL* promoter activities in human colon cancer cells.

APC^-/-^ β-catenin-TCF signaling pathways specifically enhance expression in genes containing the TCF/LEF-1 transcription factor elements. To examine the role of TBEs within the *FasL* promoter, we generated four promoter reporter constructs. As shown in Fig. 4D, simultaneous mutations in both TBE1 and TBE2 (TBE1M/2M) significantly decreased *FasL* promoter activities in SW480 cells. However, *FasL* promoter constructs containing either one mutant TBE (single mutant TBE1  =  TBE1M/2W or single mutant TBE2  =  TBE1W/2M) failed to affect promoter activities in SW480 cells (Fig. 4D), suggesting that a single TBE (TBE1 or TBE2) may be necessary and sufficient to support *FasL* promoter function in APC^-/-^ colon cancer cells.

### Effect of TBEs on FasL promoter activities in human T cells

**Figure 5:**
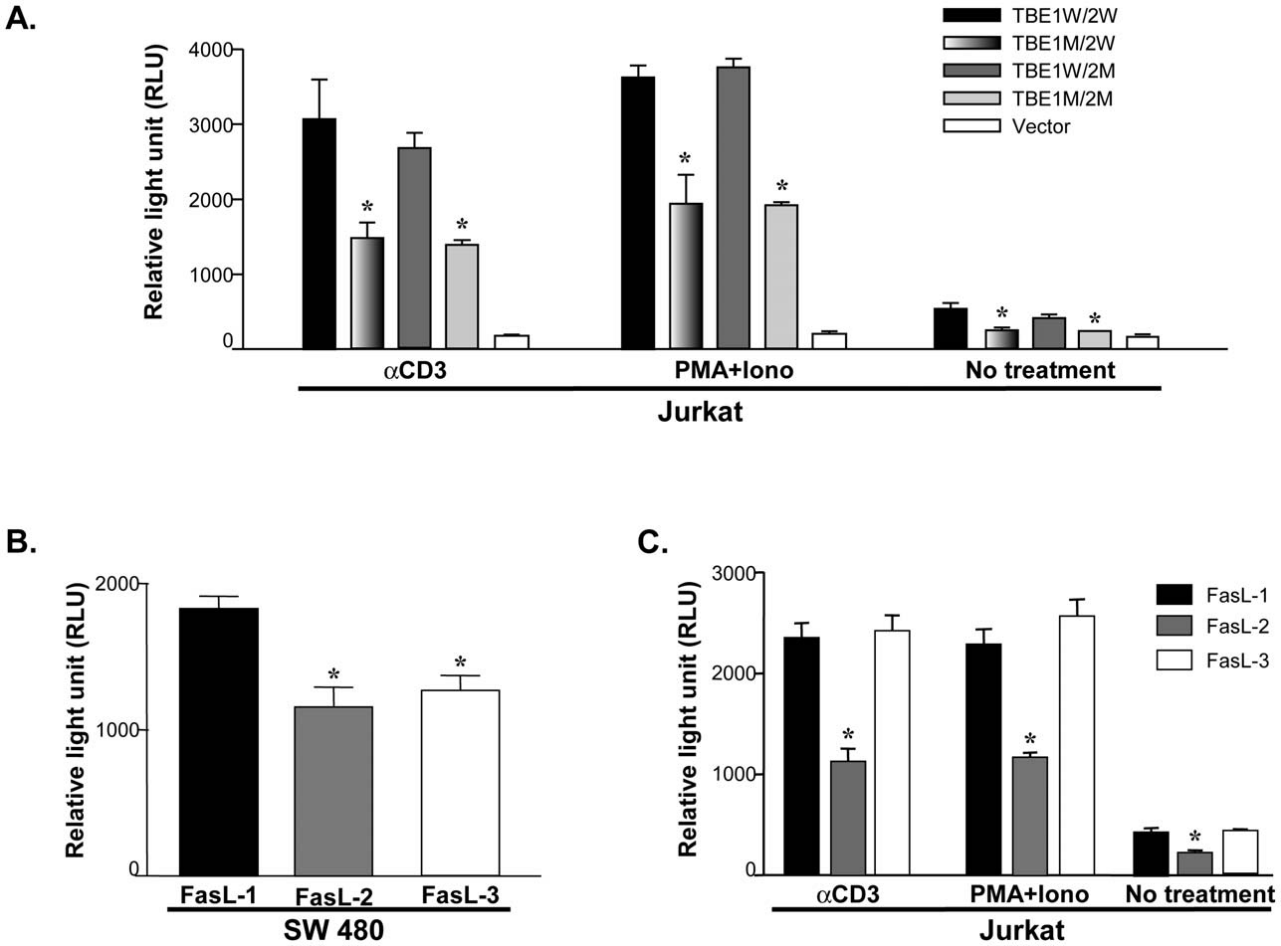
Differential regulation of *FasL* promoter activities in human T cells and in human colon cancer cells. **A**). Effect of TBE mutations on *FasL* promoter activities in Jurkat T cells. Promoter reporter construct DNA (2 µg) was transfected into Jurkat T cells as described in “Materials and Methods”. *FasL* promoter containing mutant TBE1 (TBE1M/2W) had significantly lower activities than the wild-type *FasL* promoter (TBE1W/2W) (*P<0.01). *FasL* promoter containing mutant TBE2 (-205G allele) (TBE1W/2M) did not significantly affect promoter activities compared to the wild-type reporter construct (TBE1W/2W) in Jurkat T cells. *FasL* promoter carrying both mutant TBE1 and mutant TBE2 (TBE1M/2M) also had significantly decreased promoter activity as compared with wild-type *FasL* promoter (TBE1W/2W) (*P<0.01). Data represent means ± SEM from four independent experiments. **B.**) *FasL* promoter activities of three major haplotypes in colon cancer cells. *FasL* promoter reporter constructs (0.5 µg) were transfected into SW480 cells. Both the FasL-2 and FasL-3 haplotypes had significantly lower activities compared to that of FasL-1 in colon cancer cells (*P<0.05). **C**). *FasL* promoter activities of three major haplotypes in human T cells. Promoter reporter construct DNA (2 µg) was transfected into Jurkat T cells as described in “Materials and Methods”. There were no significant differences in promoter activities between FasL-3 and FasL-1 in Jurkat T cells under various conditions (basal and stimulations with anti-CD3 or PMA plus Ionomycin). FasL-2 had significantly lower promoter activities compared to FasL-1 and FasL-3 in Jurkat T cells (*P<0.05). All relative luciferase light units (RLU) were standardized with β-galactosidase activities.

β-catenin is critical in the TCR signaling pathway which mediates TCR−CD3-driven signals necessary for T cell differentiation [48]. We speculated that TBE may be one of critical transcription elements affecting *FasL* promoter function in human T cells. In deed, as shown in Fig. 5A, *FasL* promoter carrying mutant TBE1 and wild-type TBE2 (TBE1M/2W) had significantly lower activities than that carrying both wild-type TBEs (TBE1W/2W) in T cells at all conditions (*P*<0.01). Surprisingly, *FasL* promoter carrying the wild-type TBE1 and mutant TBE2 (TBE1M/2W) failed to affect the *FasL* promoter activities in T cells (Fig. 5A). Furthermore, although mutations in both TBE1 and TBE2 (TBE1M/2M) significantly reduced *FasL* promoter activities in T cells, yet similar promoter activities were observed between the *FasL* promoter carrying mutant TBE1 with wild-type TBE2 (TBE1M/2W) and the *FasL* promoter carrying mutant TBE1 with mutant TBE2 (TBE1M/2M). Collectively, our data demonstrate that TBE1 is one of critical elements in *FasL* promoter. On the other hand, *FasL* TBE2 may not be functionally important in T cells (Fig. 5A).

### Differential effect of FasL SNP haplotpes in colon cancer cells and T cells

Single TBE1 mutation dramatically reduced *FasL* promoter activities in human T cells and but not in human colon cancer cells (Fig. 5A and Fig. 4D). Because lymphocytes (T cells) and epithelial cells (colon cancer cells) express completely different sets of transcription factors, we speculated that *FasL* promoter SNP haplotypes may function differently in T cells and colon cancer cells. In deed, as shown in Fig. 5B, FasL-3 haplotype (-844T/-756G/-478A/-205G) had significantly reduced promoter activities compared to FasL-1 (-844C/-756A/-478A/-205C) in colon cancer cells (Fig. 5B, *P*<0.05). In contrast, the promoter activities were not significantly different between FasL-3 and FasL-1 haplotypes in T cells (Fig. 5C). On the other hand, FasL-1 (-844C/-756A/-478A/-205C) always drives the highest promoter activities and FasL-2 (-844T/-756A/-478A/-205C) the lowest among the three major haplotypes in colon cancer cells and in T cells (Fig. 5B and 5C). Our data indicate that SNP haplotypes might have different effects on *FasL* expression in different cell populations.

### Role of β-catenin in the regulation FasL expression in SW480 cells

**Figure 6:**
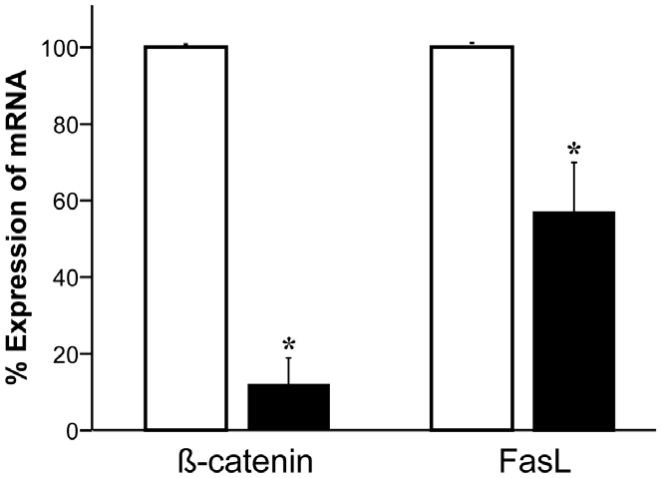
β-catenin knockdown decreases FasL expression in SW480 cells. SW480 cells were transfected with either the scrambled control siRNA (open bars) or the β-catenin siRNA (black bars) as described in “Materials and Methods). β-catenin and FasL mRNA level were calculated by relative quantification using GAPDH as the control in real-time RT-PCR assay. Introduction of β-catenin siRNA decreased 88% β-catenin and 42% FasL mRNA expression in SW480 cells (*P<0.01). Data were the average of three experiments.

To further examine whether β-catenin is involved in *FasL* expression, we utilized β-catenin siRNA to knockdown expression of β-catenin in SW480 cells. As shown in Fig. 6, β-catenin mRNA expression decreased by 88% in SW480 cells transfected with β-catenin specific siRNA as compared to the cells transfected with scrambled control siRNA. Concomitantly, the expression of *FasL* mRNA decreased by 42% in the cells along with the decreased β-catenin mRNA. These data indicate that β-catenin promotes expression of FasL.

## Discussion

TCF/LEF-1 transcription factors are expressed in lymphoid cells and cancer cells. TCF/LEF-1 transcription factor family has four major members; TCF-1, LEF-1, TCF-3, and TCF-4, which share homology in their DNA binding domain with members of the HMG box transcription factors family [49]. TCF/LEF-1 family members were originally identified as lymphoid-specific DNA-binding proteins that recognize the nucleotide sequence 5′-CTTTG(A/T)-3′ (or in reverse orientation, 5′-(A/T)CAAAG-3′) [50]–[52]. TCF/LEF-1 factors bind their target DNA sequences within gene promoters through the HMG box DNA binding domain. The HMG box not only mediates DNA sequence recognition, but also induces a dramatic bend in the DNA to facilitate assembly of functional nucleoprotein structures [53], [54]. DNA binding by TCF/LEF-1 alone is not sufficient to cause transcription activation. Promoter activation is accomplished only after a functional bipartite transcription factor is created thorough complex formation between TCF/LEF-1 transcription factor and β-catenin [55]. Within the functional complex, TCF/LEF-1 contributes the DNA binding and β-catenin confers the transcription activation [56]. Therefore, TCF/LEF-1 family factors serve as repressor to suppress target gene expression in the absence of β-catenin binding [57]. The functional transcription complexes of TCF/LEF-1 transcription factors and β-catenin are formed as a result of extracellular signal transduction in which TCF/LEF-1 factors act as molecular switches for the gene expression.

TCF/LEF-1 binds a set of target genes and programs their transcriptional competence to respond to specific signaling for β-catenin, a key down stream component of the Wnt signaling pathway. In the presence of Wnt signals, the signaling cascade leads to translocation of β-catenin into nuclei, where β-catenin interacts with TCF/LEF-1 to generate transcriptionally active multi-protein complexes [46], [49]. β-catenin is also a target of TCR signaling pathway as TCF/LEF-1 transcription factor members play critical roles in the T cell development [51], [58].

Colon cancers typically express high levels of FasL, which presumably serves as a potent mediator of immune privilege [28]. Expression of FasL potentially enables colon tumors to counterattack Fas-sensitive anti-tumor immune effector cells through Fas-mediated death. The high prevalence of expression of FasL in various tumor cells suggests that FasL may be a general perhaps essential factor in the inhibition of anti-tumor responses by cancer cells [19]–[29], [36]. Despite the tremendous attention to define FasL expression in cancer cells, it remains unclear how non-lymphoid cancer cells gain ability to express FasL and whether genetic variations affect FasL expression in cancer cells. It is well established that mutations in the adenomatous polyposis coli (APC) tumor suppressor gene initiate the majority of colorectal cancers. One consequence of this inactivation is constitutive activation of β-catenin/TCF-mediated transcription [47], [59]–[61]. Wnt pathway targets many genes critical for cell survival and proliferation (list of target genes can be found at www.stanford.edu/~rnusse/pathways/targets.html). The c-myc oncogene has been identified as a critical target gene in the Wnt signal pathway and the expression of c-myc was regulated by two TCF/LEF-1 binding elements (TBEs) in c-myc promoter [62]. In the current study, we identified two TCF/LEF-1 binding elements (TBE1 and TBE2) in human *FasL* promoter. We demonstrated that both TBE1 and TBE2 were capable of forming complexes with TCF-4 and/or TCF-4-β-catenin *in vitro* and *in vivo*. Additionally, the *FasL* TCF/LEF-1 binding element could serve as an enhancer in colon cancer cells carrying APC mutations. Mutational analysis and co-transfection assays further confirmed that TBE1 and TBE2 were involved in the regulation of *FasL* promoter activities in human cells. Furthermore, β-catenin knockdown significantly decreased FasL expression. Taken together, we propose that *FasL* is a target gene in the APC pathway and that the *FasL* TBEs are the critical transcriptional elements controlling *FasL* expression in cancer cells. Accordingly, one can speculate that the constitutively active β-catenin-TCF-4 pathways would either enhance *FasL* promoter activities in human cancer cells or enable cancer cells to express FasL through binding to *FasL* TBEs.

The identification of TCF/LEF-1 binding elements in the *FasL* promoter will provide valuable information for the understanding of FasL expression in human cancer cells. Interestingly, both TCF/LEF-1 binding elements in the *FasL* gene are either overlapped with or adjacent to known transcription factor binding sites. The TBE1 is overlapped with C/EBPβ binding motif while TBE2 is situated close to an Egr3 binding element (5′-G^-215^TGGTGT^-207^-3′) (Fig. 1C). *FasL* promoter region around SNP -205C>G contains several critical transcription elements that are essential for the optimal responsiveness to TCR-mediated activation and confer cyclosporin A sensitivity [38]-[40], [42]. Therefore, the SNP -205C>G may affect binding of multiple transcription factors to *FasL* promoter and influence the promoter activities. In the current study, we provided the direct evidence that the SNP -205C>G (in the context of haplotype) significantly affect the promoter activities in cancer cells. Additionally, SNP -205C>G has been reported to significantly associate with hemolytic anemia in lupus patients [63], suggesting that SNP -205C>G is a functional polymorphism.

Because the ability of cancer cells to express FasL may play important role in tumorigenesis, we also expanded the functional study for polymorphic C/EBPβ element (SNP -844C>T) in human colon cancer cells. We observed that the SNP in C/EBPβ also significantly altered FasL promoter activity in colon cancer cells in the context of SNP haplotypes (Fig. 5B). One can imagine that the differences in promoter activities of SNPs and/or SNP haplotypes may have a significant impact in the cancer development because the levels of FasL may affect cancer cells to establish immune privilege and metastasis. Hence, the polymorphism of C/EBPβ (-844T/C) of *FasL* promoter might have significant implication in the prognosis of cancers that rely on FasL for metastasis. In deed, SNP in *FasL* promoter was reported to be significantly associated with susceptibility to esophageal squamous-cell carcinoma and cervical cancer, underscoring the functional importance of *FasL* SNPs in the cancer development [64], [65].

Cross-linking TCR (T cell receptor) on T cells is a potent signal to upregulate FasL expression, which plays a critical role in AICD of T cells [66], [67]. It is possible that TCR signaling may upregulate FasL expression through β-catenin/TCF pathway in view of the fact that TCR signaling drives nuclear accumulation of β-catenin in human T Cells [68]. Interestingly, β-catenin nuclear accumulation also down-modulates the inhibitory isoforms of TCFs (TCF1 and LEF-1) and alters the TCF isoform balance in favor of the stimulatory TCFs that are capable of binding β-catenin in T cells [68], [69]. Consequently, TCR signaling may result in the upregulation of β-catenin/TCF target genes, which is consistent with the observations of increased FasL expression after TCR stimulation [66], [67] and consistent with the assumption that FasL is β-catenin/TCF target gene.

Activation-induced cell death (AICD) is a major mechanism to maintain immune homeostasis [66], [67], [70]. Consequently, the inducibility and level of expression of FasL under basal and induction conditions could be very crucial in maintaining immune tolerance and homeostasis. The biological importance of FasL is well established in TCR mediated AICD in lymphocyte homeostasis, in the maintenance of immune privilege within certain tissues, and in inflammations and infections. In this study, we characterized the function of three major *FasL* haplotypes, which affect the FasL promoter activities in human cancer cells and T cells. The functional characterization of *FasL* SNP haplotypes will certainly facilitate our understanding of the FasL expression in various human diseases.

## Materials and Methods

### Donors

Anti-coagulated peripheral blood was obtained from healthy normal volunteers. The human studies were reviewed and approved by the institution review board of the University of Alabama at Birmingham and all donors provided written informed consent.

### Nucleic acid isolation

Genomic DNA was isolated using the Puregene DNA isolation kit (Gentra Systems, Minneapolis, MN).

### Sequencing and amplification of FasL promoter region

The *FasL* promoter region was amplified from position -1032 to +33 with the sense primer 5′-TTA TGC CTA TAA TCC CAG CTA CTC A-3′ annealing to nucleotide position from -1032 to -1008, and anti-sense primer 5′-CTG GGG ATA TGG GTA ATT GAA G-3′ annealing to position from +12 to +33 (+1 site corresponds to the A of the ATG translation start codon). The PCR reaction was performed in a 9700 PCR System with 500 ng of DNA, 300 nM of each primer, 200 µM of dNTPs, 1.5 mM of MgCl_2_, and 2.5 U of *Taq* polymerase in a 50-µl reaction volume starting with 95°C for 5 min, 35 cycles of denaturing at 94°C for 30 s, annealing at 58°C for 45 s, extension at 72°C for 1 min with a final extension at 72°C for 7 min. All the PCR products (1065 bp) were separated on 2.5% agarose gels and purified with the QIAquick Gel Extraction Kit (QIAGEN, Valencia, CA). The purified PCR products were sequenced from both directions using BigDye terminator sequence kit on an ABI 377 Sequencer (Applied Biosystems, Inc., Foster City, CA). All new data have been deposited in GenBank SNP database as rs763110 for (SNP -844C>T), rs2021837 (SNP -756A>G), rs41309790 (SNP -478A>T), and rs74124371 (SNP -205 C>G).

### Reagents

Rabbit anti human TCF-4 polyclonal IgG was from Santa Cruz Biotechnology (Santa Cruz, CA). Mouse anti β-catenin mAb IgG was obtained from Transduction Laboratories (Lexington, KY). Transfection reagents DMRIE-C and Lipofectamine 2000 were from Invitrogen. Reagents and vectors for luciferase assays were from Promega (Madison, WI). Protease inhibitor cocktail was obtained from Roche Diagnostics (GmbH, D-68305 Mannheim, Germany).

### FasL promoter reporter constructs

The *FasL* luciferase reporter constructs were generated by cloning a *Kpn* I/*Hind* III-flanked *FasL* promoter DNA (1026 bp) fragment into pGL3-Basic vector (Promega, Madison, WI). The *Kpn* I/*Hind* III-flanked DNA products were generated by PCR amplification with human genomic DNA using upper primer 5′-GGC GGA **GGT ACC** CTA TAA TCC CAG CTA CTC AG-3′ (underlined and bold nucleotides are *Kpn* I cutting site, the primer anneals at position from -1026 to -927) and lower primer 5′-GTT CCG **AAG CTT** GGC AGC TGG TGA GTC AGG C-3′ (underlined and bold nucleotides are *Hind* III cutting site, the primer anneals at position from -19 to -1). The successive changes at nucleotide position -844, -756, -205 (TBE2), and TBE1 (-838C and -833C) were generated on reporter constructs by using QuikChange Site-Directed mutagenesis kit (Stratagene, La Jolla, CA) following the vendor's instruction. For -844T construct, sense primer 5′-AAA TGA AAA CAT TG***T*** GAA ATA CAA AGC AG-3′ and anti-sense primer 5′-CTG CTT TGT ATT TC***A*** CAA TGT TTT CAT TT-3′ were used. For -756G construct, sense primer 5′-TTA ACC TGT AA***G*** TTA TGG TGA TCG GC-3′ and anti-sense primer 5′-GCC GAT CAC CAT AA***C*** TTA CAG GTT AA-3′ were used. For -205G allele (mutant TBE2, TBE2M) construct, sense primer 5′-AGT GAG TGG GTG TTT ***G***TT TGA GAA GCA GAA-3′ and anti-sense primer 5′-TTC TGC TTC TCA AA***C*** AAA CAC CCA CTC ACT-3′ were used. For mutant TBE1 (TBE1M) construct, sense primer 5′-GCG AAA TC***C*** AAA ***C***CA GCT-3′ and anti-sense primer 5′-AGC TG***G*** TTT ***G***GA TTT CGC-3′ were used (underlined and italic letters are either natural SNPs or intentional mutations).

### Generation of the triplicate TBE2 promoter reporter constructs

Four oligos were synthesized for the generation of triplicate TBE2. For the triplicate wild-type (-205C allele) TBE2 promoter reporter construct, the sense strand oligo (5′-GGC GGA **GGT ACC** GTG GGT GTT T***C*** ^-205^T TTG AGA GTG GGT GTT T***C*** ^-205^T TTG AGA GTG GGT GTT T***C*** ^-205^T TTG AGA **GGT ACC** TAA TGA-3′) and the anti-sense strand oligo (5′-TCA TTA **GGT ACC** TCT CAA A***G*** ^-205^A AAC ACC CAC TCT CAA A***G*** ^-205^A AAC ACC CAC TCT CAA A***G*** ^-205^A AAC ACC CAC **GGT ACC** TAA TGA-3′) (underlined nucleotides are *Kpn* I cutting site and the italic nucleotide is -205G/C SNP site) were annealed to form double-stranded DNA. The double-stranded DNA was then digested with restriction enzyme *Kpn* I and cloned into pGL3-Promoter reporter vector (Promega). Similarly, the mutant (-205G allele) TBE2 construct was generated by using the sense strand oligo (5′-GGC GGA **GGT ACC** GTG GGT GTT T***G*** ^-205^T TTG AGA GTG GGT GTT T***G*** ^-205^T TTG AGA GTG GGT GTT T***G*** ^-205^T TTG AGA **GGT ACC** TAA TGA -3′) and anti-sense strand oligo (5′-TCA TTA **GGT ACC** TCT CAA A***C*** ^-205^A AAC ACC CAC TCT CAA A***C*** ^-205^A AAC ACC CAC TCT CAA A***C*** ^-205^A AAC ACC CAC **GGT ACC** TAA TGA-3′).

### Generation of the human LEF-1 and β-catenin expression constructs

The human lymphoid enhancer factor-1 (LEF-1) expression construct was generated by cloning the full length LEF-1 coding region cDNA (1200 bps) into the pcDNA3.1/HisC vector (Invitrogen). The *BamH* I/*EcoR* I-flanked LEF-1 cDNA fragment was amplified with RT-PCR from human leukocyte cDNA synthesized with the SuperScript™ Preamplification System (Invitrogen). The upper primer 5′-CCG CGT **GGA TCC** ATG CCC CAA CTC TCC GGA GGA-3′ (underlined nucleotides are *BamH* I cutting site) anneals at position from 655 to 675 and lower primer 5′-CAC GAT **GAA TTC** TCA GAT GTA GGC AGC TGT CAT-3′ (underlined nucleotides are *EcoR* I cutting site) anneals at position from 1834 to 1854 (human LEF-1 GenBank accession number: AF288571). The human β-catenin expression construct was generated by cloning *BamH* I/*Not* I-flanked β-catenin coding region cDNA (2388 bps) into pcDNA3.1 (Invitrogen). The *BamH* I/*Not* I-flanked RT-PCR products were generated with human cDNA using an upper primer (5′-CGC GGA **GGA TCC** GAA AAT CCA GCG TGG ACA ATG GCT AC-3′, underlined nucleotides are *Bam*H I cutting site) annealing at position from 197 to 222 and a lower primer (5′-AAG GAA AAA A**GC GGC CGC** CAG ACA ATA CAG CTA AAG GAT GAT-3′, underlined nucleotides are *Not* I cutting site) annealing at position from 2561 to 2584 (human β-catenin GenBank accession number: X87838). The RT-PCR reactions were carried out with KOD HiFi DNA Polymerase (Novagen, EMD Biosciences, Inc., Madison, WI) for human LEF-1 or with Expand Long Template PCR System (Roche Diagnostics GmbH, D-68305 Mannheim, Germany) for human β-catenin by following vendors' instructions.

The sequences and orientations of all cloned constructs were verified by fluorescent automated DNA sequencing from both directions on an ABI 377 Sequencer with ABI BigDye Terminator Cycle Sequencing Kit.

### Transient transfection and luciferase assays

Human colon cancer cell lines SW480 and SW620 with APC mutations (APC^-/-^) were obtained from ATCC (Manassas, VA). The cells were maintained in the L-15 medium supplemented with 10% fetal calf serum and L-glutamine (2 mM). The COS-7 cells (ATCC) were maintained in the DMEM medium supplemented with 10% fetal calf serum and L-glutamine (2 mM). The transient transfections were carried out in a 6-well tissue culture plate (Corning). The cells (2×10^5^ cells per well) were transiently transfected with 4 µl of Lipofectamin 2000 reagent (Invitrogen), 0.5 µg reporter construct plasmid DNA, and 0.05 µg pRL-null plasmid DNA (Promega) (or pCMV.SPORT-β-gal plasmid DNA, Invitrogen)) by following vendor's instruction. The transfected cells were cultured for 20 hours before being washed twice with PBS (pH 7.4). The cells were lysed in the wells with the addition of 500 µl of 1× lysis buffer for the Luciferase Assay Systems (Promega, Madison, WI). The cell supernatants were used for luciferase reporter assays by following vendor's instruction (Promega, Madison, WI).

The human leukemic T cell line (Jurkat clone E6-1, ATCC) was maintained in RPMI with 10% fetal calf serum, penicillin (1000 units/ml), streptomycin (1000 units/ml), and glutamine (2 mM). Each transient transfection experiment was carried out with 2×10^6^ Jurkat cells, 2 µg of reporter construct plasmid DNA plus 0.5 µg of pCMV.SPORT-β-gal plasmid DNA, and 4 µl of DMRIE-C reagent. Transfected cells were then cultured separately without treatment (no treatment), with treatment of 50 ng/ml PMA plus 1 μM ionomycin (PMA+Iono), or with anti-CD3 treatment (αCD3) for 18 hrs as previously described [44]. The harvested cells were washed twice with PBS (pH 7.4) before being lysed in 300 µl of 1× lysis buffer. Cell debris was removed by centrifugation and the supernatants were used in the luciferase reporter assay [44]. Relative luciferase light units, standardized to β-galactosidase activities in luciferase assays or standardized to Renilla luciferase activities in dual luciferase reporter assays, are reported as the mean of triplicate samples.

### Nuclear extract preparations

SW480 cancer cells were grown to monolayer and harvested by treating with 0.25% trypsin and 0.02% EDTA. Jurkat cells (3.5×10^6^/ml) were either stimulated with 10 µg/ml of LPS for two hours or cultured on anti-CD3 mAb coated plates for 3.5 hours before nuclear proteins were extracted as previously described [44].

### Electrophoretic mobility shift assays (EMSAs)

For each binding reaction, 8 µg of nuclear extract was incubated in 1× binding buffer (4% glycerol, 1 mM MgCl_2_, 0.5 mM EDTA, 0.5 mM DTT, 50 mM NaCl, 10 mM Tris-HCl, pH 7.5, and 50 µg/ml poly(dI-dC)^.^poly(dI-dC)) with ^32^P labeled probes in a volume of 10 µl. Binding reactions were carried out at room temperature for 30 min with 50,000 cpm (0.1–0.5 ng) of double-stranded oligonucleotides end-labeled with [γ-^32^P]ATP using T4 polynucleotide kinase. Unlabeled specific (SP) or non-specific (NP) competitor probes were used at 200-fold excess. Protein/DNA complexes and unbound DNA probe were then resolved on 5% non-denaturing polyacrylamide gel and visualized by autoradiography. The following double-stranded oligonucleotide probes were used in these experiments: 1) wild-type TBE1 (TBE1), 5′-GCG AAA TAC AAA GCA GCT-3′; mutant TBE1 (mTBE1), 5′-GCG AAA TCC AAA CCA GCT-3′ (the underlined nucleotides are introduced mutations). 2) wild-type TBE2 (-205C allele, TBE2), 5′-GTG GGT GTT TCT TTG AGA-3′; mutant TBE2 (-205G allele, mTBE2), 5′-GTG GGT GTT TGT TTG AGA-3′ (SNP -205 is underlined); nonspecific probe (NP), 5′-AAA ACA TTG TGA AAT ACA-3′. Assays with anti TCF-4 or anti β-catenin antibodies were carried out by following vendors' instruction (Santa Cruz, CA; Transduction Laboratories, Lexington, KY).

### Confirmation of the TBE/DNA bindability by the chromatin immunoprecipitation (ChIP) assay

Chromatin immunoprecipitation (ChIP) assays were performed with Chromatin Immunoprecipitation Assay Kit as described by the vendor (Upstate, Lake Placid, NY). Briefly, Jurkat T cells (1×10^7^) were washed twice with ice cold PBS. The cells were fixed and cross-linked in 1% formaldehyde. The cells were then centrifuged and washed twice with ice cold PBS containing 1× proteinase inhibitor cocktail (Roche Diagnostics GmbH, D-68305 Mannheim, Germany). Finally, the cells were lysed in SDS Lysis Buffer for 10 minutes on ice. The lysates were sonicated to shear DNA to lengths between 200 and 600 bps. The sonicated cell supernatant was diluted 10 folds in ChIP dilution buffer and pre-cleared with salmon sperm DNA/Protein A agarose beads for 30 minutes at 4°C with agitation. Anti human TCF-4 antibody was added to the pre-cleared supernatant. Salmon sperm DNA/Protein A agarose beads were used to collect the antibody/transcription complexes. The DNA extracted from the precipitated complexes was used for PCR amplification by following the vendor's instruction. For TBE1 ChIP assay, upper primer 5′-AAT AAA TAA ACT GGG CAA ACA-3′ (annealing from position -883 to -863) and lower primer 5′-AAC TAC CAT TTA CCC TGA CCT-3′ (annealing from position -741 to -721) were used. For TBE2 ChIP assay, upper primer 5′-CAG AAA ATT GTG GGC GGA AAC TT-3′ (annealing from position -291 to -269) and lower primer 5′-CGG GAC CCT GTT GCT GAC TG-3′ (annealing from position -97 to -78) were used. The ChIP assay PCR reactions were set up with 300 nM of each primer, 200 µM of dNTPs, 1.5 mM of MgCl_2_, 2.5 U of *Taq* polymerase, and 2 µl of the DNA template in a 50-µl reaction volume. The cycle reaction was started with 95°C for 5 min, 40 cycles of denaturing at 94°C for 30 s, annealing at 56°C for 45 s, extension at 72°C for 1 min with a final extension at 72°C for 7 min. The specific ChIP DNA products were separated on 2.5% agarose gels along with the 100 bp DNA molecular weight marker (Invitrogen) and the identities of PCR products of ChIP assays were further confirmed with direct DNA sequencing.

### Haplotype determination

Haplotypes were deduced from homozygous donors and by using the PHASE 2.0.1 program for haplotype reconstruction [71], [72]. Further confirmation of the *FasL* SNP haplotypes was carried out by cloning *FasL* promoter genomic DNA (1065 bps) from six heterozygous donors. The FasL promoter DNA fragment was cloned into pGEM-T Easy Vector (Promega Cor., Madison, WI) following vendor's manual. At least ten clones from each donor were sequenced on an ABI 377 Sequencer with ABI Dye Terminator Cycle Sequencing Kit.

### β-catenin knockdown and quantitative real-time RT-PCR

The human colon cancer cell line SW480 was maintained in DMEM supplemented with 10% fetal calf serum and L-glutamine (2 mM). Cells (5×10^5^) were plated in each well of 12-well plates. Cells were transfected with 50 nM (final concentration in medium) of siRNA corresponding to either β-catenin or a Scrambled control siRNA (Dharmacon, SMARTpool siRNA, ThermoFischer Lafeyette, CO) using Lipofectamine 2000 transfection reagent (Invitrogen, Carlsbad, CA) after they reached 75% confluence (approximately 24 hours after plating). Cells were harvested 48 hours after transfection and RNA was isolated using the Qiagen RNAeasy isolation system (Qiagen, MD) according to manufacturer's instructions. Purified RNA samples were subsequently treated with RNAse-free DNAse I (Invitrogen) before reverse transcription with Quantas qScript cDNA Supermix according to manufacturer's instructions (Quantas Biosciences, MD). Real-time RT-PCR was performed using Ssofast EvaGreen Supermix with low Rox (Biorad, CA) on a 7500 Real Time PCR System with 7500 Software v2.0.1 (Applied Biosytems, Foster City, CA). Melting curve analysis was performed to ensure that the primers amplified the desired amplicons and that primer dimers were absent. The following primers were used for our assay: β-catenin-Forward (5′-TCT TGC CCT TTG TCC CGC AAA TCA-3′), β-catenin-Reverse (5′-TCC ACA AAT TGC TGC TGT GTC CCA-3′), GAPDH-Forward (5′-CCT CAA CGA CCA CTT TGT-3′), GAPDH-Reverse (5′-TGG TCC AGG GGT CTT ACT-3′), FasL–Forward (5′- AAC CAA GTG GAC CTT GAG ACC ACA-3′), and FasL–Reverse (5′-TTC ACA TGG CAG CCC AGA GTT CTA-3′). Fold change in mRNA expression was calculated by relative quantification using the comparative C_T_ method with GAPDH as endogenous control. The experiments were repeated three times with triplicate samples.

### Data analysis

TESS (Transcription Element Search Software; http://www.cbil.upenn.edu/tess/) and MatInspector (http://www.gsf.de/biodv/matinspector.html) were used to search for candidate transcription factors around the SNP sites. Differences in *FasL* promoter activities of various constructs were analyzed by Student's *t* test. The null hypothesis was rejected at the 95% confidence level (*P*<0.05).
